# PRMT5 regulates colorectal cancer cell growth and EMT via EGFR/Akt/GSK3β signaling cascades

**DOI:** 10.18632/aging.202407

**Published:** 2021-01-20

**Authors:** Yongrong Yan, Peipei Zhao, Zihuan Wang, Zhen Liu, Zhizhi Wang, Jinglan Zhang, Yanqing Ding, Xing Hua, Lina Yu

**Affiliations:** 1 Department of Pathology, Nanfang Hospital and Basic Medical College, Southern Medical University, Guangzhou 510515, Guangdong Province, People’s Republic of China; 2 Guangdong Province Key Laboratory of Molecular Tumor Pathology, Guangzhou 510515, Guangdong Province, People’s Republic of China; 3 The First Clinical Medical Department, Southern Medical University, Guangzhou 510515, Guangdong Province, People’s Republic of China; 4 Department of Pathology, Guangzhou Red Cross Hospital, Jinan University, Guangzhou 510220, Guangdong Province, People’s Republic of China

**Keywords:** PRMT5, EMT, Akt, GSK3β, EGFR

## Abstract

Emerging evidence shows that type II protein arginine methyltransferase 5 (PRMT5) serves as an oncoprotein and plays a critical role in many types of human cancer. However, the precise role and function of PRMT5 in human colorectal cancer (CRC) growth and epithelial-mesenchymal transition (EMT) are still unclear, and the related molecular mechanism and signaling axis remains largely obscure. Here, we show that PRMT5 is highly expressed in CRC cell lines and tissues. Using PRMT5 stable depletion cell lines and specific inhibitor, we discover that down-regulation of PRMT5 by shRNA or inhibition of PRMT5 activity by specific inhibitor GSK591 markedly suppresses CRC cell proliferation and cell cycle progression, which is closely associated with PRMT5 enzyme activity. Moreover, PRMT5 regulates CRC cell growth and cycle progression via activation of Akt, but not through ERK1/2, PTEN, and mTOR signaling pathway. Further study shows that PRMT5 controls EMT of CRC cells by activation of EGFR/Akt/GSK3β signaling cascades. Collectively, our results reveal that PRMT5 promotes CRC cell proliferation, cell cycle progression, and EMT via regulation of EGFR/Akt/GSK3β signaling cascades. Most importantly, our findings also suggest that PRMT5 may be a potential therapeutic target for the treatment of human colorectal cancer.

## INTRODUCTION

Human colorectal cancer (CRC) is the most common tumor globally, which affects human life and results in death. CRC is a chronic disease, and the clinical manifestations are sporadic and develop very slowly in most humans over many years. The accumulating evidence suggested that in most cases, several genes mutation, including *APC*, *KRAS*, and *TP53*, was involved in the CRC development and progression [1]. Nevertheless, there are still sporadic CRC developing via different signaling axis, including oncogene *BRAF* activation and CpG methylation [2]. Thus, accurate diagnosis and detection of CRC in the early stage are incredibly urgent. However, the CRC molecular pathogenesis and related signaling pathways are heterogeneous and still obscure.

Protein arginine methyltransferases (PRMTs) have been involved in and play critical roles in multiple cellular physiological processes, including chromatin remodeling, gene expression, RNA splicing, cell cycle, cellular signaling crosstalk, proliferation, apoptosis, and protein functions [3, 4]. Protein arginine methyltransferase 5 (PRMT5), the type II protein arginine methyltransferase, catalyzes the symmetrical dimethylation of histone or non-histone protein at the arginine residues. Accumulating evidence has shown that PRMT5 is the ectopic expression in many types of human cancer and regulates cell metabolism, including lung cancer, pancreatic cancer, breast cancer, leukemia, lymphoma, glioblastoma, prostate cancer, and bladder cancer [5, 6]. Additionally, it has been reported that the PRMT5 expression level is closely associated with the low expression of E2F1 in CRC patients [7]. Moreover, PRMT5 repressed the tumor suppressor F-box and WD repeat domain containing 7 (FBW7), which led to elevating c-Myc expression levels and promoting aerobic glycolysis and cancer cell proliferation [8]. However, there is not enough evidence to elucidate the major functions of PRMT5 in CRC, and the molecular mechanism of PRMT5 in the regulation of CRC cell growth, epithelial-mesenchymal transition (EMT), and the related signaling axis is entirely unknown.

In the current study, we uncovered the novel role of PRMT5 in human colorectal cancer and revealed the underlying molecular mechanism by which PRMT5 regulated CRC cell proliferation and EMT. Our findings showed that PRMT5 was highly expressed in CRC cell lines and tissues. Moreover, PRMT5 controls cell growth and cell cycle progression via activation of Akt, which is closely related to the enzyme activity of PRMT5. Most importantly, PRMT5 gated EMT probably through EGFR/Akt/GSK3β signaling cascades.

## RESULTS

### PRMT5 is overexpressed in human colorectal cancer cells and tissues

**Figure 1:**
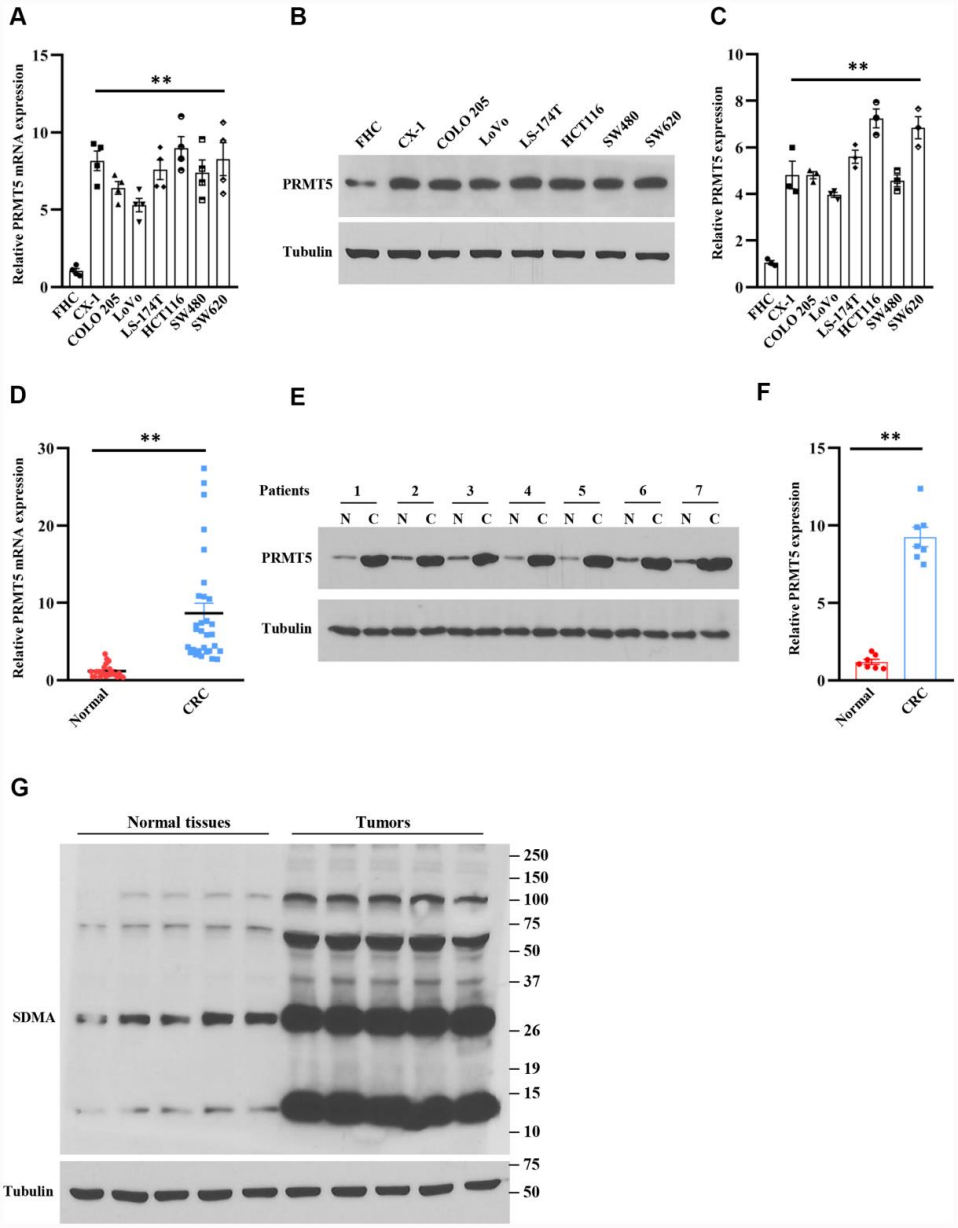
**PRMT5 is highly expressed in human colorectal cancer cells and tissues.** (**A**) The mRNA expression level of PRMT5 in various human colorectal tumor cell lines and normal colonic mucosal FHC cells (n=4). **P < 0.01 vs. FHC cells. (**B**) Western blot analysis of PRMT5 protein expression level in various human colorectal tumor cell lines and normal colonic mucosal FHC cells. Representative data is shown. (**C**) PRMT5 protein expression level is quantified. **P < 0.01 vs. FHC cells. (**D**) qRT-PCR analysis of PRMT5 mRNA expression level in human colorectal cancer tissues and adjacent normal tissues (n=25-30). **P < 0.01 vs. normal tissues. (**E**) PRMT5 protein expression level is analyzed by Western blotting in human colorectal cancer tissues and adjacent normal tissues (n=7). Representative data is shown. (**F**) PRMT5 protein expression level is quantified. **P < 0.01 vs. normal tissues. (**G**) The global symmetric dimethylarginine (SDMA) is detected by Western blotting. Representative data is shown.

A previous study has shown that PRMT5 is involved in many types of human cancer and tumor progression, including colorectal cancer [9]. In order to explore the function of PRMT5 in human colorectal cancer, we firstly evaluated the mRNA expression level of PRMT5 in various human colorectal cancer cell lines. As shown in Figure 1A, PRMT5 mRNA expression level was markedly increased in those colorectal cancer cell lines compared with normal colonic mucosal FHC cells. Furthermore, we detected the PRMT5 protein expression level using the same cell lines. As shown in Figure 1B, 1C, the PRMT5 protein expression level was dramatically increased compared to the normal colonic mucosal FHC cells, indicating that PRMT5 is highly expressed in human colorectal cancer cells. To further confirm that PRMT5 has participated in human colorectal cancer, we collected human colorectal tumors and adjacent normal tissues from patients. As shown in Figure 1D, the PRMT5 mRNA expression level was significantly elevated in colorectal tissues compared with normal tissues. Moreover, Western blot analysis showed that PRMT5 protein expression level was distinctly elevated as well (Figure 1E, 1F), implying that PRMT5 plays an essential role in human colorectal cancer development and progression.

Arginine methylation plays a key role in the regulation of gene transcription and post-translational protein modification [4]. Moreover, most of the cellular symmetric di-methylarginine (SDMA) is produced by PRMT5. Therefore, the enzymatic activity of PRMT5 can be assessed by global SDMA. Next, we further evaluated whether PRMT5 was engaged in colorectal cancer. As shown in Figure 1G, the SDMA was markedly increased in colorectal tumor tissues compared with normal tissues. Altogether, our findings indicate that PRMT5 is an ectopic expression in human colorectal cancer, and the PRMT5 enzyme activity is closely linked to colorectal cancer.

### Down-regulation of PRMT5 suppresses cell proliferation and cell cycle progression

**Figure 2:**
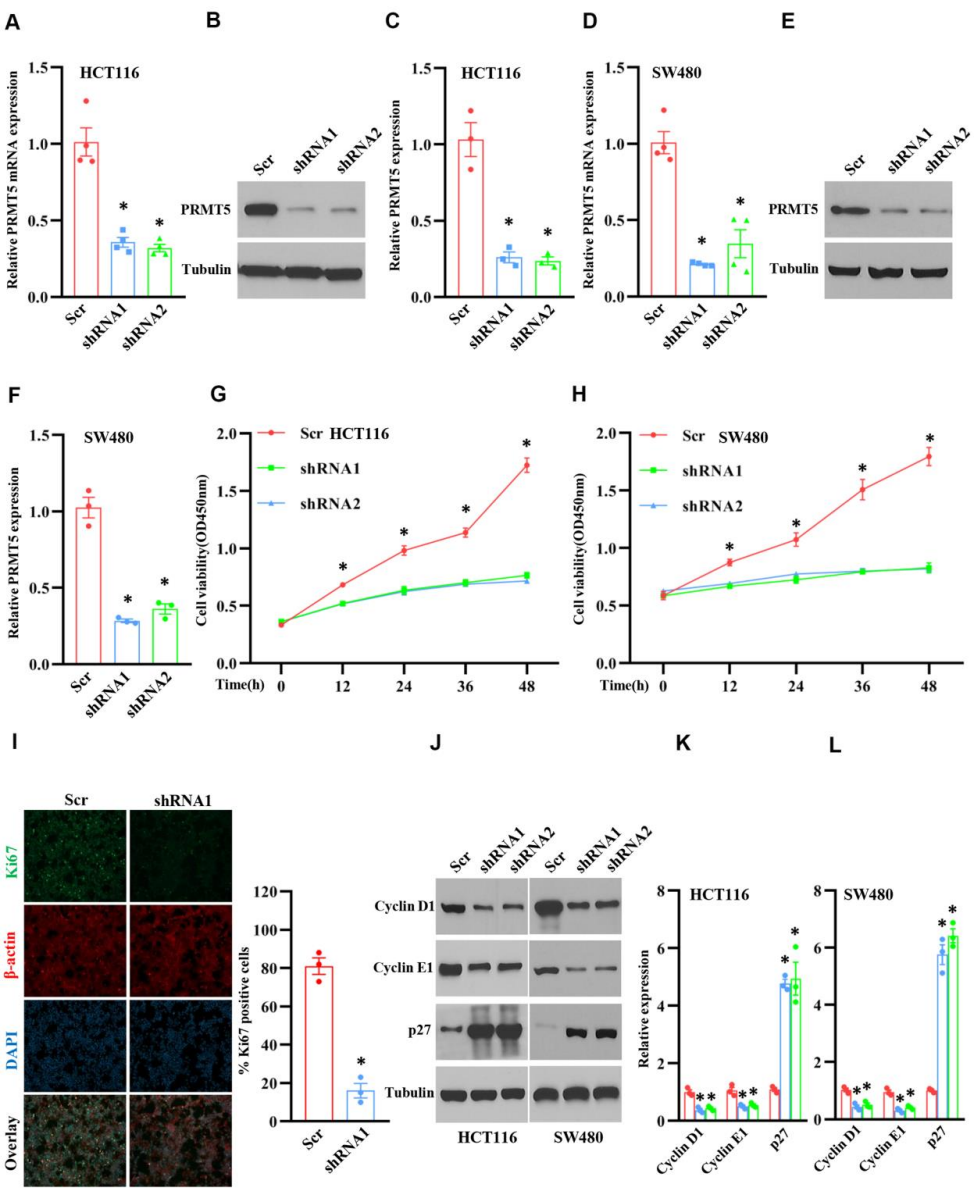
**Silencing PRMT5 prevents cell proliferation and cell cycle progression.** HCT116 and SW480 cells are transduced by lentivirus containing Scramble and PRMT5-shRNAs. (**A**) qRT-PCR analysis of PRMT5 mRNA expression level in HCT116 cells. *P < 0.05 vs. Scramble (Scr). (**B**) PRMT5 protein expression level is detected by Western blotting in HCT116 cells. Representative data is shown. (**C**) PRMT5 protein expression level is quantified in HCT116 cells. *P < 0.05 vs. Scr. (**D**) qRT-PCR analysis of PRMT5 mRNA expression level in SW480 cells. * *P* < 0.05 vs. Scr. (**E**) PRMT5 protein expression level is analyzed by Western blotting in SW480 cells. (**F**) PRMT5 protein expression level is quantified in SW480 cells. *P < 0.05 vs. Scr. (**G**, **H**) Cell viability is measured during different time points in HCT116 and SW480 cells (n=4). *P < 0.05 vs. Scr. (**I**) Immunostaining of ki67, a cell proliferation marker, in HCT116 cells. Representative pictures were shown. Scale bar = 50μm. The Ki67 positive cells were quantified in the indicated groups. For each group, 1000 cells were counted. n=3, *P < 0.05 vs. Scr. (**J**) Western blot analysis of cyclin D1, cyclin E1, and p27 protein expression level in HCT116 and SW480 cells. Representative data is shown. (**K**, **L**) Indicated proteins are quantified in HCT116 and SW480 cells. *P < 0.05 vs. Scr. Red bar = Scr, Blue bar = shRNA1, and Green bar = shRNA2.

In order to explore the role of PRMT5 in human colorectal cancer, we generated the PRMT5 stable knockdown cell lines using lentivirus containing PRMT5 shRNAs and Scramble (Scr). As shown in Figure 2A, 2D, PRMT5 mRNA expression level was significantly reduced in HCT116 and SW480 cells compared to Scr. Western blot analysis further showed that PRMT5 protein expression level was distinctly impaired in HCT116 and SW480 cells as well (Figure 2B, 2C, 2E, 2F). Subsequently, those PRMT5 depletion cell lines were applied to cell viability assay. As shown in Figure 2G, 2H, the cell viability was dramatically prevented at different time points when PRMT5 was down-regulated in HCT1116 and SW480 cells. Moreover, we also assessed cell proliferation using ki67, a well-known cell proliferation marker. As shown in Figure 2I, ki67 positive cells were markedly reduced when PRMT5 is silenced in HCT116 cells. These results suggest that PRMT5 regulates colorectal cancer proliferation. Next, we asked if PRMT5 could control the cell cycle progression. In this regard, we detected the related cell cycle markers, including cyclin D1, cyclinE1, and cell cycle inhibitor p27. As shown in Figure 2J–2L, we found that cyclin D1 and cyclin E1 protein expression level was significantly lower in HCT116 and SW480 cells compared with Scr when PRMT5 is down-regulated. In contrast, the p27 protein expression was remarkably elevated in HCT116 and SW480 cells compared with Scr. Collectively, our findings indicate that PRMT5 control human colorectal cancer cell growth and cell cycle progression.

### Inhibition of PRMT5 blocks cell growth and cell cycle progression

**Figure 3:**
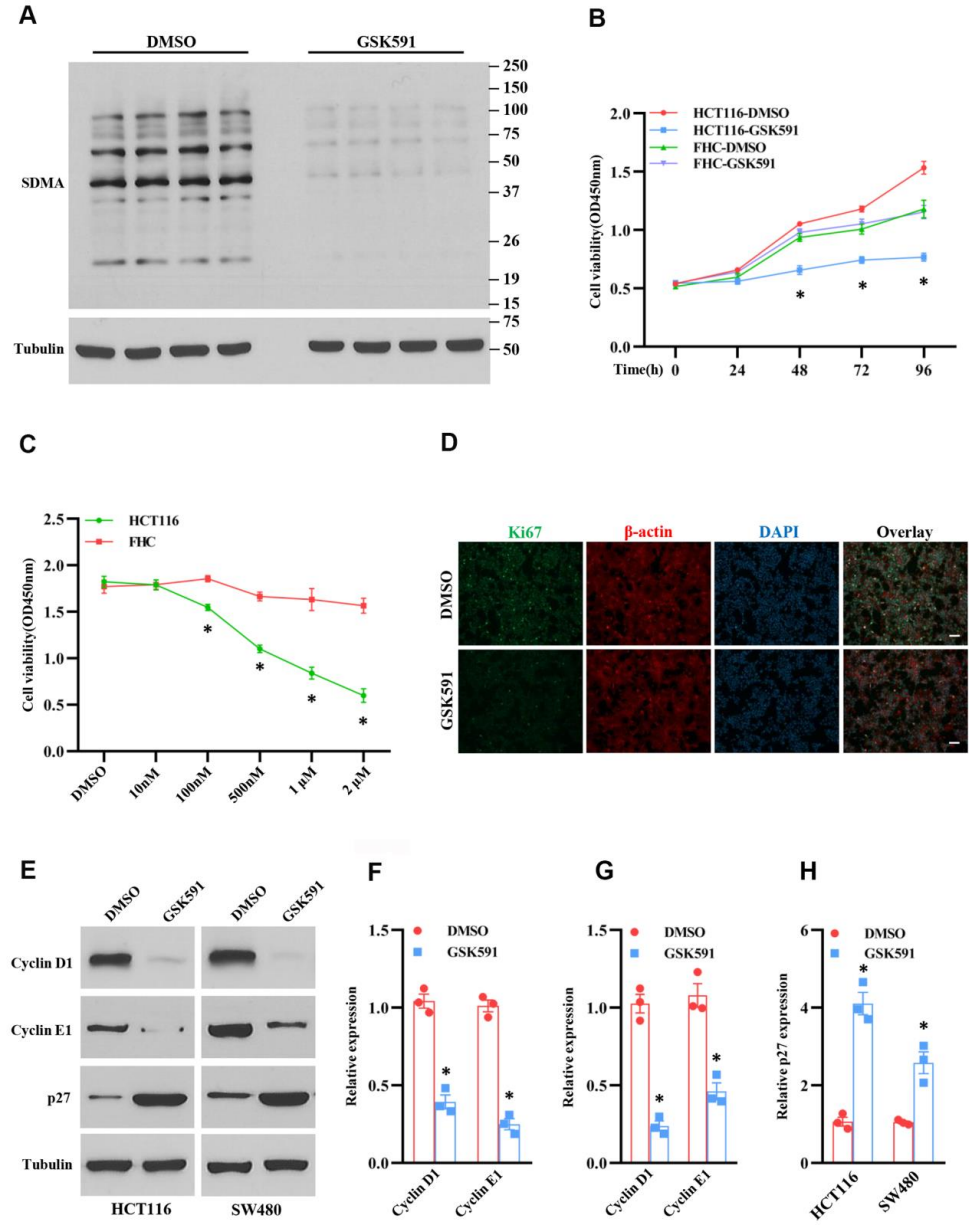
**Inhibition of PRMT5 suppresses cell growth and cell cycle progression.** HCT116, SW480, and FHC cells are treated with different doses and time points of GSK591, a PRMT5 specific inhibitor. (**A**) The global symmetric dimethylarginine (SDMA) is detected by Western blotting. Representative data is shown. (**B**) Cell viability is measured during different time points in HCT116 and FHC cells (n=4). *P < 0.05 vs. DMSO treatment. (**C**) Cell viability is measured in HCT116 and FHC cells with different doses of GSK591 (n=4). *P < 0.05 vs. FHC cells. (**D**) Immunostaining of ki67 in HCT116 cells treated with GSK591. Scale bar = 50μm. (**E**) Western blot analysis of cyclin D1, cyclin E1, and p27 protein expression level in HCT116 and SW480 cells. Representative data is shown. (**F**–**H**) Indicated proteins are quantified in HCT116 and SW480 cells. *P < 0.05 vs. DMSO.

PRMT5 regulated gene expression and protein post-translational modification through methylation [10]. Therefore, the enzyme activity of PRMT5 plays a crucial role in the regulation of DNA and protein methylation. To further investigate the enzyme activity of PRMT5 was required or not for the colorectal cancer cell proliferation, we used GSK591, a PRMT5 specific inhibitor, to treat HCT116 cells, and the SDMA expression was assessed. As shown in Figure 3A, the SDMA expression level was almost diminished compared to the DMSO treatment, indicating that GSK591 completely blocked the enzyme activity of PRMT5. Next, we treated the HCT116 and FHC cells with GSK591, and the cell viability was monitored at different time points. As shown in Figure 3B, GSK591 distinctly prevented HCT116 cell growth compared with DMSO treatment. By contrast, GSK591 did not affect the cell growth of FHC cells. Furthermore, we found that GSK591 blocked HCT116 cell growth in a dose-dependent manner (Figure 3C). To further confirm our findings above, we performed immunostaining for ki67 in HCT116 cells treated with GSK591. As shown in Figure 3D, cell proliferation was almost suppressed in HCT 116 cells compared with DMSO treatment. These results indicate that the enzyme activity of PRMT5 is required for colorectal cancer cell proliferation. Next, we asked if the enzyme activity of PRMT5 could be required for the cell cycle progression. To this end, we evaluated the cyclin D1, cyclinE1, and p27 expression levels in HCT116 and SW480 cells treated with GSK591. As shown in Figure 3E–3H, cyclin D1 and cyclinE1 protein expression levels were almost completely blocked in HCT116 and SW480 cells compared with DMSO treatment. In contrast, the p27 protein expression was remarkably elevated in HCT116 and SW480 cells compared with DMSO treatment. Taken together, our results suggest that the enzyme activity of PRMT5 is required for colorectal cancer cell proliferation and cell cycle progression.

### PRMT5 regulates colorectal cancer cell growth via Akt activation

**Figure 4:**
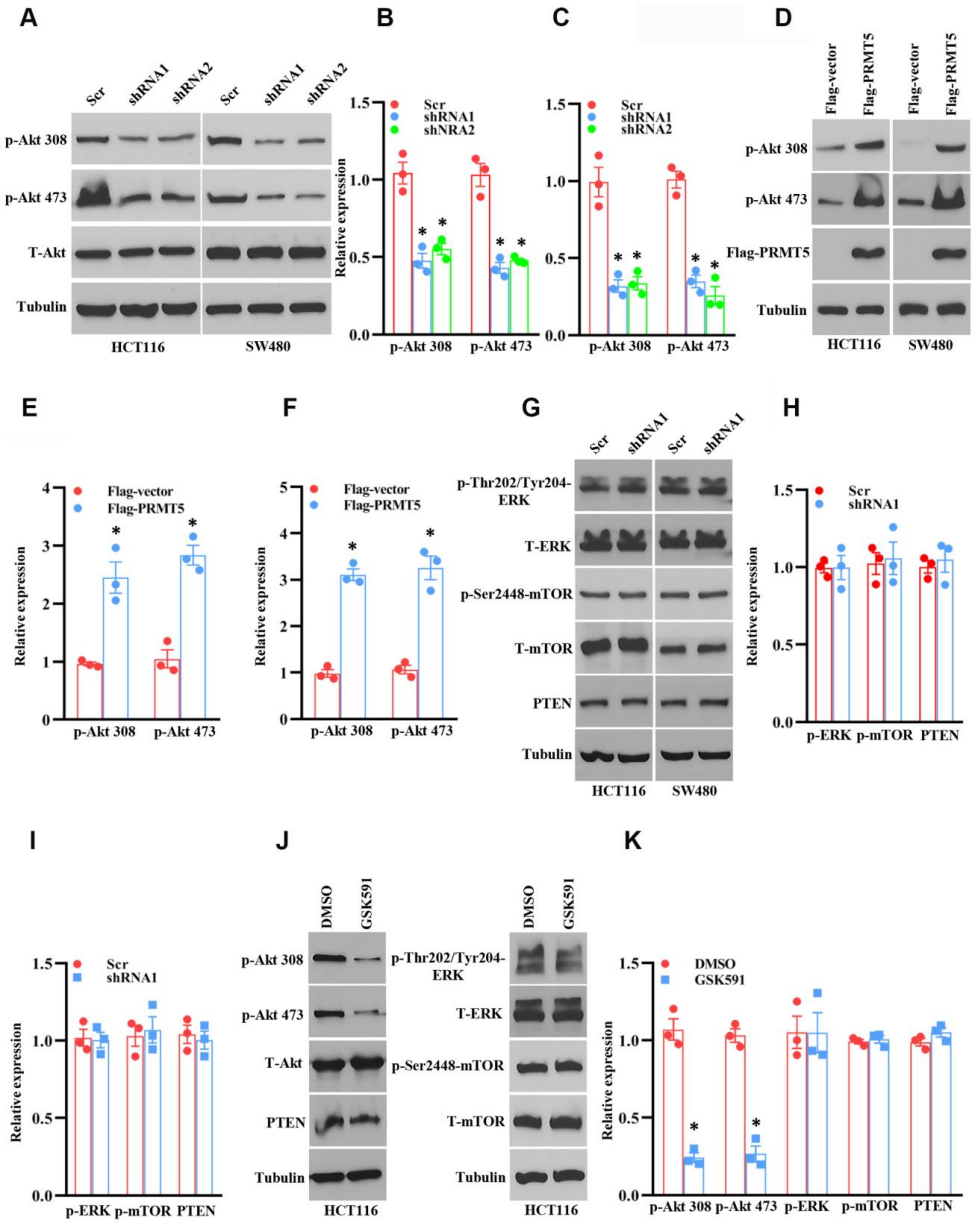
**PRMT5 regulates Akt activation in human colorectal cancer cells.** (**A**) Western blot analysis of phospho-Akt and total Akt protein expression level in HCT116 and SW480 cells. Representative data is shown. (**B**, **C**) The indicated proteins are quantified in HCT116 and SW480 cells. *P < 0.05 vs. Scr. (**D**) Western blot analysis of indicated protein expression level in HCT116 and SW480 cells. Representative data is shown. (**E**, **F**) The indicated proteins are quantified in HCT116 and SW480 cells. *P < 0.05 vs. Flag vector. (**G**) Western blot analysis of indicated protein expression level in HCT116 and SW480 cells. Representative data is shown. (**H**, **I**) The indicated proteins are quantified in HCT116 and SW480 cells. (**J**) Western blot analysis of indicated protein expression level in HCT116 cells. Representative data is shown. (**K**) The indicated proteins are quantified in HCT116 cells. *P < 0.05 vs. DMSO.

Although PRMT5 is participated in human colorectal cancer, how PRMT5 gates the tumor cell growth and epithelial-mesenchymal transition (EMT) and the related molecular mechanisms are still entirely unknown. To this end, we assessed the protein kinase B (also named Akt) activity, which is a master regulator for tumor cell proliferation and cell cycle progression [11]. As shown in Figure 4A–4C, the phospho-Thr308 and phospho-Ser473 Akt expression level was remarkably decreased when PRMT5 was down-regulated in HCT116 and SW480 cells, implying that PRMT5 controlled colorectal cancer cell proliferation and cell cycle progression through regulation of Akt activity. To further confirm our hypothesis, we re-introduce PRMT5 into the PRMT5 depletion cells. As shown in Figure 4D–4F, overexpression of PRMT5 recovered the phospho-Thr308 and phospho-Ser473 Akt expression level in HCT116 and SW480 cells. These results indicate that PRMT5 regulates Akt activity and colorectal cancer cell growth. It has been reported that PRMT5 regulates colorectal cancer cell growth via ERK signal cascades [9]. Nevertheless, we did not see any changes in the phospho-ERK1/2 expression when PRMT5 was down-regulated in HCT116 and SW480 cells (Figure 4G, 4H). Additionally, we detected the phospho-mTOR and PTEN in PRMT5 depilation cell lines as well. We found that silencing PRMT5 did not affect the expression level of phospho-mTOR and PTEN, which regulated Akt activation (Figure 4G, 4H). These results suggest that PRMT5 regulates Akt activation independent ERK1/2, PTEN, and mTOR.

Next, we asked if the enzyme activity of PRMT5 is associated with Akt activation. In order to test this hypothesis, HCT116 cells were treated with PRMT5 inhibitor GSK591, and the phospho-Akt was detected by Western blotting. As shown in Figure 4J, 4K, we found that the phospho-Thr308 and phospho-Ser473 Akt expression level was significantly reduced compared with DMSO treatment, but total Akt expression did not change. Besides, we did not see any changes in the expression of phospho-ERK1/2, phospho-mTOR, and PTEN, suggesting that the enzyme activity of PRMT5 is closely linked to the Akt activity. Collectively, our findings uncover that PRMT5 controls human colorectal cancer cell proliferation via activation of Akt, but not ERK1/2 or PTEN/mTOR signaling pathways.

### PRMT5 regulates EMT via EGFR/Akt/GSK3β signaling axis

**Figure 5:**
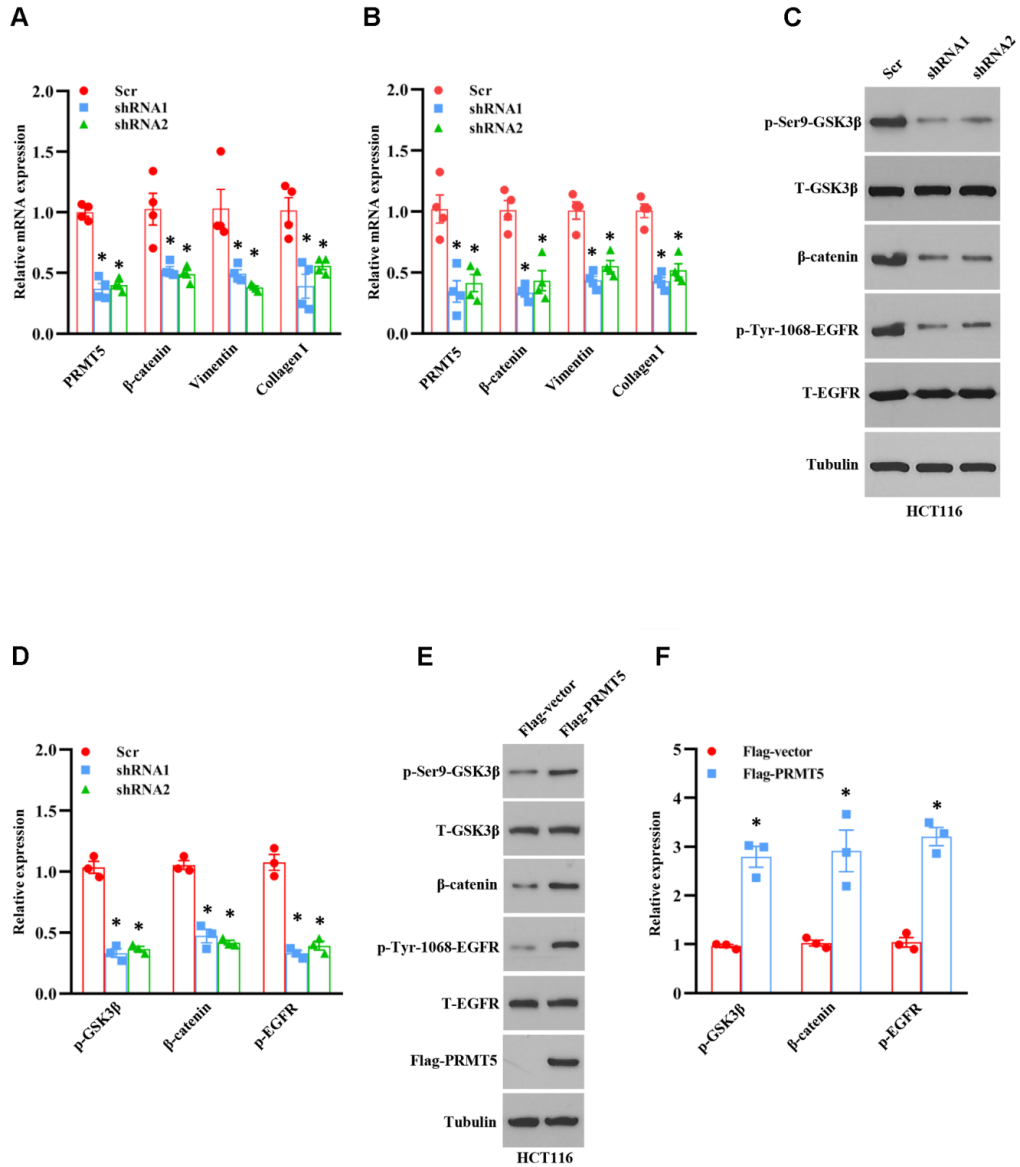
**PRMT5 control EMT through Akt/GSK3β signaling axis.** (**A**, **B**) qRT-PCR analysis of EMT markers in HCT116 and SW480 cells. *P < 0.05 vs. Scr. (**C**) Western blot analysis of indicated protein expression level in HCT116 cells. Representative data is shown. (**D**) The indicated proteins are quantified in HCT116 cells. *P < 0.05 vs. Scr. (**E**) Western blot analysis of indicated protein expression level in HCT116 cells. Representative data is shown. (**F**) The indicated proteins are quantified in HCT116 cells. *P < 0.05 vs. DMSO.

The emerging evidence has been shown that Akt/EMT signaling axis plays a vital role in human colorectal cancer progression [12]. Nevertheless, the upstream target of these signaling is still unknown and not well characterized. According to the above results, we wondered whether PRMT5 was involved in the EMT via activation of Akt in colorectal cancer. To this end, the EMT markers were measured by qRT-PCR using PRMT5 depletion cell lines. As shown in Figure 5A, 5B, the mRNA expression level of β-catenin, vimentin, and collagen I was dramatically reduced in HCT116 and SW480 cells when PRMT5 was down-regulated by shRNA, indicating that PRMT5 is engaged in EMT progression. GSK3β is the most critical downstream target of Akt and controls β-catenin stability, which is involved in EMT [13, 14]. To further investigate the function of PRMT5, we detected the protein expression level of phospho-GSK3β and β-catenin by Western blotting in PRMT5 silencing cells. As shown in Figure 5C, 5D, we found that phospho-GSK3β and the β-catenin protein expression level was markedly decreased, whereas the total GSK3β was unchanged. These results indicate that PRMT5 regulates EMT through Akt/GSK3β signaling in colorectal cancer. Epidermal growth factor receptor (EGFR) acts as a receptor tyrosine kinase (RTK) that regulates cell growth, metabolism, invasion, migration, survival, and EMT via activation of the PI3K/Akt signaling pathway [15, 16]. A recent study has been reported that PRMT5 promotes EMT via EGFR in pancreatic cancer cells [17]. We further evaluate the EGFR expression in PRMT5 silencing cells. We found that phospho-EGFR was significantly impaired, whereas the total EGFR was unchanged (Figure 5C, 5D), suggesting that PRMT5 regulates EGFR activation in colorectal cancer. In order to confirm our results, we re-introduce the PRMT5 into the PRMT5 silencing cells. We found that the phospho-GSK3β, β-catenin, and phospho-EGFR were recovered in HCT116 cells (Figure 5E, 5F), indicating that PRMT5 indeed regulates EGFR/Akt/GSK3β signaling cascades. Altogether, our findings reveal that PRMT5 controls EMT through activation of the EGFR/Akt/GSK3β signaling pathway in colorectal cancer cells.

## DISCUSSION

So far, it has been reported that PRMT5 acts as an oncoprotein and plays a crucial role in different types of human cancer via regulation of specific downstream targets or signaling cascades. Nevertheless, it is completely unknown whether PRMT5 is engaged in human colorectal cancer cell proliferation and EMT. Besides, the related molecular mechanism is also unclear. In the current study, we confirm that PRMT5 is frequently highly expressed in human colorectal cancer cells and tissues in both mRNA and protein levels (Figure 1). Further studies show that silencing PRMT5 by shRNA or inhibition of PRMT5 by specific inhibitor GSK591 suppresses colorectal cancer cell growth and cell cycle progression, which is closely related to PRMT5 enzyme activity (Figures 2, 3). Moreover, PRMT5 controls human colorectal cancer cell proliferation via the activation of Akt, but not ERK1/2 or PTEN/mTOR signaling pathways (Figure 4). Finally, our findings reveal that PRMT5 promotes EMT through activation of the EGFR/Akt/GSK3β signaling pathway in colorectal cancer cells (Figure 5). These results suggest that PRMT5 expression level, enzyme activity, and its downstream targets could be served as the potential therapeutic candidates for the treatment of human colorectal cancer. Our findings also pose the possibility that PRMT5 promotes colorectal cancer cell growth, cell cycle progression, and EMT via EGFR/Akt/GSK3β signaling axis.

The oncoprotein PRMT5 promotes cancer cell proliferation, tumorigenesis, tumor invasion, and metastasis, which has been well elucidated in many human cancers. For instance, PRMT5 epigenetically activates the transcription of the androgen receptor and facilitates prostate cancer cell growth [18]. PRMT5 also regulates FBW7 expression and EGFR/β-catenin signaling pathway to promote pancreatic cancer proliferation and tumorigenesis [8, 17]. A recent study has been reported that PRMT5 co-localizes with Akt and regulates Akt activation, which accelerates human lung cancer cell growth [19]. Additionally, the down-regulation of PRMT5 prevents cell proliferation at the G1 phase in human lung cancer [20]. The transcriptional factor E2F1, a master regulator for cell cycle, controls cell entry from G1 to S phase during cell cycle progression [7]. A previous study has shown that PRMT5 directly methylated E2F1 and regulates the cell cycle, which is required for gating the function of E2F1, suggesting that E2F1 methylation by PRMT5 leads to cancer cell cycle progression. All the above observations indicate that PRMT5 plays a pivotal role in cancer cell proliferation and cell cycle progression and that PRMT5 is a key upstream regulator for cancer cell growth. In the present study, we showed that PRMT5 not only overexpressed in colorectal cancer cells and tissues (Figure 1), but also regulated cell cycle progression and promoted cell growth via regulation of cyclin D1, cyclin E1, and p27, using PRMT5 stable knockdown cell lines and specific inhibitor GSK591 (Figures 2, 3), which was associated with PRMT5 enzyme activity. Furthermore, the accumulated evidence showed that Akt/GSK3β signaling pathway had participated in control the expression of cyclin D1 and cyclin E1 in different cell types [21, 22]. It is possible that PRMT5 regulates cyclin D1 and cyclin E1 through regulation of the Akt/GSK3β signaling axis (Figure 4). These data strongly suggest that PRMT5 can serve as an oncoprotein and a vital effector in carcinogenesis and progression of human colorectal cancer.

**Figure 6:**
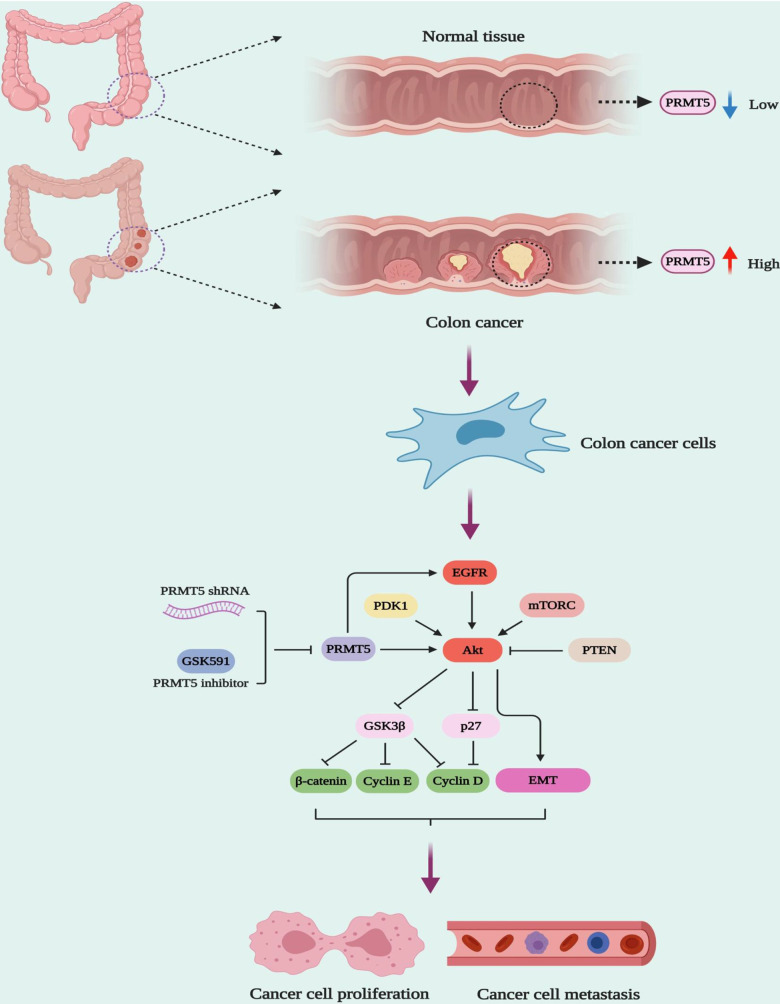
**The model of PRMT5 regulation of human colorectal cancer cell growth and EMT.**

The PI3K-Akt signaling pathway is responsible for cell growth, cell survival, cell metabolism, cell cycle, gene expression, cell development, and anti-apoptosis in various cell types [11]. It has been shown that the dysfunction of PI3K-Akt signaling is involved in different human cancers, metabolic diseases, autoimmune disorders, nerve disease, and cardiovascular disorders [11]. Besides, the PI3K/Akt signaling pathway plays a vital role in cancer cell proliferation and EMT, which promotes drug resistance, metastasis, and poor prognosis [23]. Although previous studies have shown that PRMT5 may regulate Akt activity via control of PI3K hyperphosphorylation or PTEN hypophosphorylation in cancer cells [24], how PRMT5 promotes colorectal cancer cell growth and EMT and the underlying molecular mechanism is still undiscovered. To date, on the other hand, there is no clear evidence that PRMT5 and its enzyme activity regulates Akt activity to modulate colorectal cancer cell proliferation and EMT, although it has been reported that overexpression of PRMT5 promoted Akt activation (phosphorylation) in 293T cells [24]. In our study, we show that PRMT5 could activate Akt in colorectal cancer cells independent of ERK1/2, PTEN, and mTOR signaling, using PRMT5 stable knockdown cell lines and specific inhibitor GSK591, which is required for PRMT5 enzyme activity (Figure 4). Moreover, a previous study has shown that the depletion of PRMT5 significantly reduces the expression of phospho-ERK1/2 and phospho-mTOR in colorectal cancer cells [9]. However, in our study, we did not see any changes in the expression level of phospho-ERK1/2, phospho-mTOR, total ERK, and total mTOR using PRMT5 stable depletion cell lines. It is implied that PRMT5 promotes colorectal cancer cells growth and EMT via activation of Akt, but not ERK and mTOR signaling pathway. Furthermore, it is well-established that EGFR is implicated in the regulation of cancer cell growth and tumor development [25]. A previous study has been shown that PRMT5 regulates the autophosphorylation of EGFR at Tyr1068 and Tyr1172 to promotes EMT in pancreatic cancer cells [17]. In our study, we show that PRMT5 not only regulates Akt/GSK3β activity but also control EGFR activation and EMT related gene expression (Figure 5). More importantly, our results also uncover that PRMT5 promotes EMT in colorectal cancer cells via EGFR/Akt/GSK3β signaling axis (Figure 6).

In summary, our study provides evidence that PRMT5 is overexpressed in human colorectal cancer cells and tissues. Our study also proves that PRMT5 promotes cell proliferation and EMT in human colorectal cancer via activation of the EGFR/Akt/GSK3β signaling axis, but not ERK1/2 or PTEN/mTOR signaling pathways, which is strongly linked to the PRMT5 enzyme activity. Our results further imply that PRMT5 may act as a novel therapeutic candidate for the treatment of human colorectal cancer. More importantly, the new insights into the activation of EGFR/Akt/GSK3β signaling axis medicated by PRMT5 provide much new information for the mechanisms of the carcinogenic effect of PRMT5 in human colorectal cancer.

## MATERIALS AND METHODS

### Human CRC cell culture and collection of CRC tissue samples

All human colorectal cancer cell lines, including HCT116, SW480, CX-1, SW620, LoVo, COLO 205, LS-174T, and normal colonic mucosal FHC cells, were purchased from American Type Culture Collection (ATCC; Manassas, VA, USA). The HCT116 cells were grown in DMEM/F12 containing 10% fetal bovine serum (FBS); the SW480, SW620, and LoVo cells were cultured in DMEM supplemented with 10% FBS; the CX-1 and COLO 205 cells were grown in RPMI-1640 medium containing 10% FBS; the LS-174T were cultured in Eagle’s MEM containing 1% non-essential amino acids solution, 1 mM sodium pyruvate, and 10% FBS. The cells were maintained at 37° C under 5% CO_2_. The fresh human CRC samples and matched adjacent normal tissues were obtained between 2017 and 2019 from the Department of Pathology, Southern Medical University. The human samples were obtained from the patients with informed consent, and the Ethics Committee approved the current project of Southern Medical University.

### Plasmids and chemicals

Human PRMT5 knockdown lentiviral plasmids were as described previously [19]. The packaging plasmid MD2G and helper plasmid PAX2 were purchased from Addgene. The PRMT5 knockdown targeting sequences are listed below: shRNA1, 5’-GGATAAAGCTGTATGCTGT-3’; shRNA2: 5’-GCCATCTATAAATGTCTGCTA-3’. PRMT5 specific inhibitor GSK591 (cat# SML-1751) was purchased from Sigma. Human *PRMT5* cDNA subcloned into Flag vector was purchased from Addgene and verified by sequencing.

### Gene expression analysis

Total RNA was isolated from HCT116, SW480, CX-1, SW620, LoVo, COLO 205, LS-174T, and FHC cells and the human CRC and normal tissues using TRIzol reagent (Cat# 15596-018; Invitrogen) according to the manufacturer’s protocol. Subsequently, an equal amount of RNA was applied to reverse transcription using Bio-Red PCR thermal cycler (C1000). For gene expression analysis, the SYBR green fluorescent Dye (cat# 1725272; Bio-Rad) was subjected to the quantitative real-time PCR (qRT-PCR) with an ABI7500 PCR machine (Applied Biosystems). The following primers were used: human PRMT5 forward: 5’-CCTGTGGAGGTGAACACAGT-3’ and revise: 5’-AGAGGATGGGAAACCATGAG-3’; GAPDH, forward: 5’-GAAGGTGAAGGTCGGAGTCAACG-3’ and revise: 5’-TGCCATGGGTGGAATCATATTGG-3’; β-catenin forward: 5’-AAAGCGGCTGTTAGTCACTGG-3’ and revise: 5’-CGAGTCATTGCATACTGTCCAT-3’; Vimentin forward: 5’-AGTCCACTGAGTACCGGAGAC-3’ and revise: 5’-CATTTCACGCATCTGGCGTTC-3’;

Collagen I forward: 5’-GAGGGCCAAGACGAAGACATC-3’ and revise: 5’-CAGATCACGTCATCGCACAAC-3. GAPGH was served as an internal control. Relative mRNA expression was determined by the method of ΔΔ-Ct.

### Lentivirus preparation and PRMT5 stable knockdown cell line generation

Lentivirus containing PRMT5-shRNAs was prepared by co-transfection along with the packaging plasmid MD2G and helper plasmid PAX2 into 293T cells. The medium was harvested after 48h, and the viral titer was pre-determined. The equal amount of virus particles was used in the indicated experiments. In order to generate the PRMT5 stable knockdown cells, the HCT116 and SW480 cells were infected with the lentivirus containing PRMT5-shRNAs or scramble shRNA. Those cells were then selected with puromycin (1 μg/mL, cat# p9620; Sigma) for 48 h, and the non-infected cells were killed. Finally, the PRMT5-depletion stable cells were used for the indicated experiments.

### Cell transfection

In order to deliver the exogenous genes into cells, the Lipofectamine^TM^ 3000 (cat#L3000015, Invitrogen) was used. Before transfection, the HCT116 and SW480 cells were seeded into 6-well plates. When the cells were grown around 70%, the plasmids of flag-vector and flag-PRMT5 was delivered into those cells. After 48h, the cells were harvested for Western blotting.

### Western blotting

Western blotting was carried out as described previously [26]. Briefly, proteins were extracted from indicated cell lines using the lysis buffer (20 mmol/L Tris, PH 7.4, 2 mmol/L EDTA, 2 mmol/L EGTA, 1 mmol/L sodium orthovanadate, 1% Triton X-100, 150 mmol/L NaCl, 50 mmol/L sodium fluoride, 0.1% SDS and 100 mmol/L phenylmethylsulfonyl fluoride) and separated in sodium dodecyl sulphate/polyacrylamide gel electrophoresis (SDS/PAGE). The proteins were transferred to the PVDF membranes (cat#1620177; Bio-Rad), and the membranes were washed three times with TBST for 10 min at room temperature. Subsequently, the membranes were incubated with indicated antibodies as follows: PRMT5 (cat# sc-376937; Santa Cruz Biotechnology), cyclin D1 (cat# 2978; Cell Signaling Technology), cyclin E1 (cat# 20808; Cell Signaling Technology), P27 (cat# 3686; Cell Signaling Technology), phospho-Thr308-Akt (cat# 13038; Cell Signaling Technology), phospho-Ser473-Akt (cat# 4060; Cell Signaling Technology), total Akt (cat# 4691; Cell Signaling Technology), phospho-Ser9-GSK3β (cat# 5558; Cell Signaling Technology), total GSK3β (cat# 12456; Cell Signaling Technology), phospho-ERK1/2 (cat# 4370; Cell Signaling Technology), total ERK1/2 (cat# 4695; Cell Signaling Technology), phospho-Ser2448-mTOR (cat# 5536; Cell Signaling Technology), total mTOR (cat# 2983; Cell Signaling Technology), PTEN (cat# 9188; Cell Signaling Technology), Symmetric Di-Methyl Arginine Motif [sdme-RG] MultiMab™ (cat# 13222; Cell Signaling Technology), phospho-Tyr-1068-EGFR (cat# 3777; Cell Signaling Technology) total EGFR (cat# 2085; Cell Signaling Technology), β-catenin (cat# 8480; Cell Signaling Technology) and β-tubulin (cat# 5666; Cell Signaling Technology). The membranes were washed and labeled with goat anti-rabbit conjugated to HRP secondary antibody (cat# sc-2004 Santa Cruz Biotechnology) or goat anti-mouse conjugated to HRP secondary antibody (cat# sc-2005; Santa Cruz Biotechnology). Immunoreactivity was determined by SuperSignal West Pico Chemiluminescent Substrate Western blotting detection reagents (cat# 34580; Thermo Fisher Scientific).

### Cell viability assay

HCT116 and SW480 cells were transduced with lentivirus containing scramble shRNA and PRMT5 shRNAs and seeded into 24-well plates (5000 cells/plate). Cell viability was detected by cell counting kit-8 (CCK-8; Dojindo Molecular Technologies, Rockville, MD, USA) during the different time points according to the manufacturer’s recommendation. The Infinite 200 plate reader (TECAN, Mönnedorf, Switzerland) was applied to evaluate cell viability. In order to assess the effect of PRMT5 inhibitor GSK591 on cell viability, HCT116 and SW480 cells were seeded into 96-well plates (5000 cells/plate) and treated with DMSO and different concentrations of GSK591.

### Immunofluorescence

HCT116 cells were seeded into 6-well cell culture plates and incubated overnight to establish adherence. The cells were fixed with 3.7% paraformaldehyde for 15 minutes at room temperature, followed by permeabilization in ice-cold methanol for 15 minutes at -20^o^ C. Subsequently, the cells were incubated with blocking buffer (PBS containing 5% normal goat serum and 0.5% Triton X-100) for 1 hour at room temperature, followed by incubation with Ki67 (cat# ab209897, Abcam) and β-actin (cat# sc-47778, Santa Cruz Biotechnology) antibody (diluted 1:500 in blocking buffer) at 4° C overnight. Next, the cells were washed three with PBS for 10 minutes, and then incubated with Alexa Fluor 488-conjugated goat anti-rabbit secondary antibody (cat# A-11034; Thermo Fisher) for Ki67 and Alexa Fluor 594-conjugated goat anti-mouse secondary antibody (cat# A-11004; Thermo Fisher) for β-actin (diluted 1:500 in blocking buffer) and at room temperature. Nuclei were stained with DAPI (cat# D9542; Sigma) for 30 minutes at room temperature before observation. The Nikon Eclipse E800 fluorescence microscope was used to capture the images.

### Statistical analysis

Unless stated otherwise, the data were represented as means ± SEM. Paired and unpaired two-tailed Student’s *t*-test was used for comparisons with the indicated group. The difference with *P*<0.05 was considered statistically significant. For all Figures, *P< 0.05 and **P < 0.01 vs. indicated group.
